# Power, potential, and pitfalls in global health academic partnerships: review and reflections on an approach in Nepal

**DOI:** 10.1080/16549716.2017.1367161

**Published:** 2017-09-15

**Authors:** David Citrin, Stephen Mehanni, Bibhav Acharya, Lena Wong, Isha Nirola, Rekha Sherchan, Bikash Gauchan, Khem Bahadur Karki, Dipendra Raman Singh, Sriram Shamasunder, Phuoc Le, Dan Schwarz, Ryan Schwarz, Binod Dangal, Santosh Kumar Dhungana, Sheela Maru, Ramesh Mahar, Poshan Thapa, Anant Raut, Mukesh Adhikari, Indira Basnett, Shankar Prasad Kaluanee, Grace Deukmedjian, Scott Halliday, Duncan Maru

**Affiliations:** ^a^ Possible, Kathmandu, Nepal; ^b^ Department of Anthropology, University of Washington, Seattle, WA, USA; ^c^ Department of Global Health, University of Washington, Seattle, WA, USA; ^d^ Henry M. Jackson School of International Studies, University of Washington, Seattle, WA, USA; ^e^ Health Equity Action Leadership Initiative, University of California, San Francisco, San Francisco, CA, USA; ^f^ Division of Hospital Medicine, University of California, San Francisco, San Francisco, CA, USA; ^g^ Gallup Indian Medical Center, Gallup, NM, USA; ^h^ Department of Psychiatry, University of California, San Francisco, San Francisco, CA, USA; ^i^ Shared Minds, Boston, MA, USA; ^j^ Tuba City Regional Health Care, Tuba City, AZ, USA; ^k^ Nepal Health Research Council, Ministry of Health, Kathmandu, Nepal; ^l^ Public Health Monitoring & Evaluation Division, Ministry of Health, Kathmandu, Nepal; ^m^ Department of Medicine, Division of Global Health Equity, Brigham and Women’s Hospital, Boston, MA, USA; ^n^ Department of Medicine, Division of General Pediatrics, Boston Children’s Hospital, Boston, MA, USA; ^o^ Department of Medicine, Division of General Internal Medicine, Massachusetts General Hospital, Boston, MA, USA; ^p^ Department of Medicine, Harvard Medical School, Boston, MA, USA; ^q^ Department of Obstetrics and Gynecology, Boston Medical Center, Boston, MA, USA; ^r^ Department of Obstetrics and Gynecology, Boston University School of Medicine, Boston, MA, USA; ^s^ Department Medicine, Division of Women’s Health, Brigham and Women’s Hospital, Boston, MA, USA; ^t^ District Health Office, Department of Health Services, Ministry of Health, Mangalsen, Achham, Nepal; ^u^ School of Leadership and Development, Eastern University, St. Davids, PA, USA; ^v^ Tséhootsooí Medical Center, Fort Defiance, AZ, USA; ^w^ Department of Global Health and Social Medicine, Harvard Medical School, Boston, MA, USA

**Keywords:** Global health, partnership, Nepal, health equity, training support

## Abstract

**Background**: Global health academic partnerships are centered around a core tension: they often mirror or reproduce the very cross-national inequities they seek to alleviate. On the one hand, they risk worsening power dynamics that perpetuate health disparities; on the other, they form an essential response to the need for healthcare resources to reach marginalized populations across the globe.

**Objectives**: This study characterizes the broader landscape of global health academic partnerships, including challenges to developing ethical, equitable, and sustainable models. It then lays out guiding principles of the specific partnership approach, and considers how lessons learned might be applied in other resource-limited settings.

**Methods**: The experience of a partnership between the Ministry of Health in Nepal, the non-profit healthcare provider *Possible*, and the Health Equity Action and Leadership Initiative at the University of California, San Francisco School of Medicine was reviewed. The quality and effectiveness of the partnership was assessed using the Tropical Health and Education Trust Principles of Partnership framework.

**Results**: Various strategies can be taken by partnerships to better align the perspectives of patients and public sector providers with those of expatriate physicians. Actions can also be taken to bring greater equity to the wealth and power gaps inherent within global health academic partnerships.

**Conclusions**: This study provides recommendations gleaned from the analysis, with an aim towards both future refinement of the partnership and broader applications of its lessons and principles. It specifically highlights the importance of targeted engagements with academic medical centers and the need for efficient organizational work-flow practices. It considers how to both prioritize national and host institution goals, and meet the career development needs of global health clinicians.

## Background

### Foundations in inequities

At their core, global health academic partnerships (GHAPs) involve complex and dynamic, typically unequal power relationships between nations, people, and institutions [1,2]. GHAPs can help leverage resources such as funding, professional development opportunities, and clinical and research expertise from places of privilege and power in high-income countries (HICs) to improve healthcare delivery and systems design in low- and middle-income countries (LMICs) [3–9]. Similarly, there is increasing recognition of LMICs as drivers of innovation [10–13], and the personal and professional benefits that accrue to participants from HICs have been well documented [14–16].

Getting these partnerships ‘wrong’, however, can have grave consequences, such as iatrogenic harm, medicalization of poverty, the undermining and fragmentation of national healthcare systems through uncoordinated efforts, and the perpetuation of global north–global south exploitative relations [17–21]. Fostering impactful, ethical, and equitable engagements that strengthen national capacity and delivery systems in LMICs is challenging, precisely because these partnerships are often forged across staggering inequalities [1,22].

A growing body of literature, broad in scope and disciplinary focus, examines the assemblages of actors from rich and poor countries that make up GHAPs [23]: these include universities, international/non-governmental organizations, external donor partners, philanthropic foundations, research groups, and government bodies such as Ministries of Health. Many articles reveal the ethical dilemmas and potential unintended consequences – as well as the blurred definitional and operational boundaries – of short-term medical missions [24,25], global health electives [26,27], and medical volunteer trips [28–32]. Others seek to delineate qualities of effective global health partnerships [33], create principles and codes of ethical engagement [19,34–39] that may guide the creation of ethically sound and successful partnerships, or facilitate the improvement of existing ones [40].

Partnership is an increasingly amorphous term in global health and itself in need of scrutiny. The term ‘partnership’ can be misused as an almost ideological construct or panacea. We prefer to use the term as a generative and dynamic concept [41] that holds the potential to further projects for equity and social justice.

To hone in on the specific dynamics involved, we describe a partnership between the Ministry of Health (MoH) in Nepal, the non-profit healthcare provider *Possible*, and the Health Equity Action and Leadership Initiative (HEAL) at the University of California, San Francisco School of Medicine. The partnership was formed with the explicit purposes of: (1) strengthening public sector healthcare systems and national capacity through education and mentorship of LMIC-based healthcare workers, (2) serving patients in marginalized communities, (3) avoiding the pitfalls of ‘medical voluntourism’ and other forms of intermittent or inconsistent care [42], and (4) creating mutually beneficial opportunities for professional development and personal growth for both visiting and local team members.

We begin by briefly discussing the broader landscape of GHAPs, summarizing some of the key challenges to developing ethical, equitable, and sustainable models from the perspectives of host institutions and global health clinicians (GHCs). We then lay out guiding principles that animate the Nepal partnership’s approach to GHAPs, and discuss its experience over the last 2 years. We assess the quality and effectiveness of the partnership utilizing the Tropical Health and Education Trust (THET) Principles of Partnership framework [39], and discuss strengths and limitations of the partnership. We conclude with considering how lessons learned might be applied to create effective and equitable GHAPs in other resource-limited settings.

### Framing the landscape of global health academic partnerships: opportunities and challenges

While there remains no standard definition of ‘global health’ [18], efforts have been made to move towards a common definition [43]. Key features include the incorporation of research and practice with a priority of achieving health equity worldwide, and a recognition of the need for an inter-disciplinary, multi-directional approach that reaches beyond the health sciences to address these inequalities [43]. The contemporary state of our world remains characterized both by a deep interconnectedness and by marked inequalities in health and wellbeing within and between countries. The world’s interconnectedness and inequality is a necessary departure point to discuss the challenges at the center of many GHAPs.

Academic partnerships can help to address global inequalities by promoting research and advocacy to support progressive policy agendas [44], to advance medical education [9], and to retain healthcare workers in rural and other settings of economic marginalization [14,45]. Healthcare delivery organizations in LMICs can benefit from financial and human resources from which they are typically excluded. Both sending and host institutions use partnerships as a tool for recruitment and retention [15,45,46]. Healthcare workers in host institutions benefit from knowledge and skills transfer, as well as the support of being connected to an academic environment, which can attenuate some of the difficulties of working in isolation [3]. GHCs benefit from opportunities not readily available at their home site, as well as a chance to align their work with their ideals [47].

Despite these potential benefits, it is challenging for host institutions to meaningfully engage academic clinicians to leverage their expertise in an effective yet empowering way. Most global health work is part-time and intermittent by nature, due to clinical, research, and administrative responsibilities at academic centers. In a survey of US post-residency clinicians with global health work experience, 73% reported spending < 10% of professional time on global health in the prior year [48]. As such, GHCs have the potential to contribute to ‘ephemeral healthcare’, where fleeting forms of service delivery are not integrated within longitudinal systems [42]. The objectives of GHCs and their funding sources do not often align with the greatest needs of local communities [49,50], and the sustainability of GHAPs is challenging when relying on GHCs for their time commitment and efforts to fund themselves.

Existing global power structures place academic institutions, and especially physicians, at the top of power hierarchies. Due to the often irregular and brief nature of these engagements, partnerships featuring these GHCs have the potential to undermine the autonomy and agency of local staff and leadership [20,21]. The one-sided nature of travel and opportunities in most academic partnerships risk further exacerbating inequalities among global healthcare workers [27]. Advocating for reciprocity can partially mediate this concern [18,51], although bi-directional exchange requires additional resources, and, in many cases, travel opportunities are further complicated by inequalities in global visa systems. Without an orientation to local culture, language, and the plural healthcare practices of communities, it is difficult for GHCs to provide value [20,52]. An additional burden is often placed on local hosts to transport, house, feed, and translate for GHCs, thereby potentially sidelining their own areas of responsibility in favor of the interests and projects of visiting clinicians [53].

From the perspective of GHCs, it is challenging to build a productive career in global health. Global health priorities are typically not aligned with current pathways for compensation and promotion [48,54]. In one survey of hospitalists with ongoing global health work, 78% reported receiving no institutional funding, and experienced an overall lack of mentorship, support, or recognition for their global health work [55]. Family life also leads to conflicting priorities, and is an important consideration for academic clinicians [48]. Without a viable, professional career pathway, GHCs compelled by global health often fall into volunteer endeavors. As such, the medical voluntourism model tends to be the default approach to global health work, despite a growing awareness of the accompanying problems of a lack of durability, effectiveness, and accountability [42,56]. Below we discuss our efforts to create a partnership attendant to the perils of this model.

## Review

### Organizational context

The MoH directly provides essential public health and primary healthcare services throughout all 75 districts in Nepal. In a healthcare economy that has increasingly become dominated by fee-for-service for-profit private providers [57], the MoH has started to look towards public–private partnerships (PPPs) to strengthen the public sector. *Possible* is a non-profit healthcare provider that has pioneered an approach with the MoH, through which the government provides facilities, staff, supplies, and co-financing, and *Possible* assumes management authority and is held accountable for direct healthcare delivery within government infrastructure. The partnership is deeply sensitive to the contentious nature of PPPs in the arena of global healthcare delivery [58,59], and the shared goal is to both meet public sector delivery gaps in rural areas and provide a platform for public sector innovation. *Possible* targets an annual per capita price point of $US25, based upon the current levels of public sector financing in Nepal. Through the partnership, *Possible* has treated over 500,000 patients since clinical services began in 2008, and now averages over 130,000 treated per year.

*Possible* employs over 280 staff in Nepal, all of whom are Nepali citizens. A sister US-based non-profit provides technical and financial support. No expatriates are involved in clinical care in *Possible*’s model, since expatriate clinicians would never affordably be financed by the Nepali government. In addition to meeting both service delivery and innovation gaps, *Possible* aims to strengthen public sector human resource capacity. Most mid-level providers (e.g. health assistants, community medical assistants, staff nurses, auxiliary nurse midwives, pharmacists, and laboratory staff) are from the local areas, and if they leave *Possible*, tend to transition via the *lok sevā* – the Nepali public service commission. Physicians tend to be recruited from urban areas and, thus, are more transitory. After working with *Possible* for a period of 1–2 years, some seek residency training in the US, a phenomenon on the rise with recent batches of Nepali medical graduates [60]. Others transition to national post-graduate programs or directly into the public service.

HEAL is a global health equity fellowship based at the University of California, San Francisco with the goal of creating, scaling, and sustaining a pipeline of HIC- and LMIC-based healthcare professionals to care and advocate for poor and marginalized communities in the US and abroad. Essential to HEAL’s mission is to promote two-way dialogue and exchange, and to make more equitable partnerships that directly confront typical power dynamics between HIC and LMIC institutions. As part of its initiative, HEAL currently places rotating fellows in 2-year engagements, divided between foreign placements (Haiti, India, Liberia, Malawi, Mali, Mexico, and Nepal) and marginalized communities in the US. Rotating fellows are paired with site fellows from their respective communities.

Over the last 3 years, together the MoH, *Possible*, and HEAL initiated an exchange involving expatriate GHCs and Nepali staff. The program had its roots in long-standing relationships between several of the co-authors from HEAL and from *Possible* (in particular co-authors PL and DM did residency together). HEAL was also looking for new partners to iterate on the notion of partnership. *Possible*’s leadership, particularly its Nepali physician staff, ultimately decided that working with HEAL would help to bring global perspectives and knowledge to their clinical care. Vital to *Possible*’s own view of HEAL’s value proposition was that Nepali staff members would receive international opportunities. The MoH had long supported expatriate clinician-educators from *Possible* to augment national capacity, contingent on the mandate that they would not be displacing Nepali jobs and followed all legal frameworks. All of us involved in the conceptualization and implementation of the partnership brought extensive experiences in global health and had seen many of the pitfalls and power dynamics at play. Indeed, core to the founding and evolution of this partnership were our own prior mistakes. Below we explore the guiding principles and lessons learnt over the years of our collective work.

### Recruitment and management systems

The partnership currently brings on three GHCs, known as HEAL fellows, every year for 2-year commitments. HEAL fellows are screened and selected based on their experience and qualifications as they relate to organizational priorities, and a commitment to strengthening healthcare delivery systems through clinical education and mentorship. Additionally, HEAL fellow candidates are assessed by their motivation to contribute to global health equity, respect for local priorities, alignment with *Possible*’s culture code, and commitment to cultural humility. Supplemental Table 1 includes the rubric HEAL uses to assess prospective GHCs and assess the right fit of the applicant for their international placement, and a rubric used by *Possible* to assess a prospective GHC’s fit with the MoH and *Possible*’s PPP model. HEAL fellows spend roughly 50% of their professional time working with *Possible*, and 50% working clinically in a marginalized area in the US. They are early career clinicians, typically 1–2 years out of residency or fellowship training.

**Table 1.** Nepal partnership model within the THET principles of partnership framework.

Strategic Health partnerships have a shared vision, have long-term aims and measurable plans for achieving them, and work within a jointly-agreed framework of priorities and direction.	Formal engagements maintained with academic medical centers. Targeted recruitment of academic clinicians and post-graduate fellows. Measurable objectives reviewed on a quarterly basis. Managers ensure strategic mission of organization is prioritized.
Harmonized & Aligned Health partnerships’ work is consistent with local and national plans, and complements the activities of other development partners.	Structured orientation to organization’s goals, management systems, and communication tools. Managers collaborate to align goals with organizational objectives. Partnership objectives designed to influence national priorities by integrating closely with the MoH.
Effective & Sustainable Health partnerships operate in a way that delivers high-quality projects that meet targets and achieve long-term results.	Partnership duration of 2 years is targeted. Diverse funding sources including academic institutions and research grants. Internal metrics of effectiveness applied, along with site evaluation tools designed for GHCs.
Respectful & Reciprocal Health partnerships listen to one another and plan, implement, and learn together.	Bidirectional exchange with career development opportunities for Nepali staff. Faculty coaches address the career development needs of academic partners.
Organized & Accountable Health partnerships are well-structured, well-managed, efficient, and have clear and transparent decision-making processes.	Formal memorandum of understanding addresses management structure, lines of accountability, and individual responsibilities. Structured workflows facilitate orientation, travel, lodging, visa, and medical licensing. GHCs participate in organizational performance evaluations.
Responsible Health partnerships activities are conducted with integrity and cultivate trust in their interactions with stakeholders.	Strong organizational culture code reinforced with academic partners. Managers ensure GHC’s presence is empowering rather than undermining for full-time staff and leadership.
Flexible, Resourceful, & Innovative Health partnerships proactively adapt and respond to altered circumstances and embrace change.	GHCs are introduced to all organizational departments to facilitate interdisciplinary connections. Details of each partnership negotiated individually to allow for flexibility.
Committed to Joint Learning Health partnerships' stakeholders monitor, evaluate, and reflect on their activities and results, articulating lessons learned, and sharing knowledge with others.	Opportunities for reflection and feedback are structured into meetings with managers and faculty coaches. Results of partnership shared organizationally via a newsfeed. Results shared publicly through media, academic conferences, and open-access, peer-reviewed publications.

*Possible* seeks to integrate GHCs fully into organizational systems and structures, and especially its mission of public sector strengthening. GHCs receive a structured ‘onboarding’ to the organization’s culture code, goals, management systems, and project management tools. GHCs are also introduced to all organizational departments to facilitate interdisciplinary connections and break down ‘siloed’ working environments. GHCs are encouraged to learn deeply about Nepal, including socio-demographic and health trends, historical and contemporary patterns of structural violence, national and regional political economies, agricultural and changing dietary patterns, and plural systems of healing. Cultural and language resources are provided, with recognition that mastering the Nepali language for a 2-year fellowship is an unreasonable expectation.

As part of the integration process, GHCs are assigned a direct manager and a faculty coach. Direct managers are the Nepali medical director at the GHC’s site who conduct weekly structured meetings, develop quarterly work plans, and conduct biannual performance evaluations akin to full-time employees. They aim to ensure GHC expertise is leveraged in an effective and empowering way, in which full-time staff view these engagements as a resource rather than a burden. Faculty coaches have a funded engagement with *Possible*, and an academic appointment at a US university. They meet monthly to support GHCs, with a goal of ensuring accountability, and that the strategic mission of the organization is being prioritized. Roles of the coach include support for research and writing, and attention to career development needs. These are unique given that expectations for promotion and departmental funding may be outside the expertise of the manager. Included in the supplementary materials is an organizational protocol detailing the division of labor between manager and faculty coach, which helps ensure these roles leverage each other effectively (Supplemental Table 2). An example of a structured meeting template and a set of GHC areas of responsibilities are provided in Supplemental Files 1 and 2, respectively.

### Scope of work by global health clinicians

GHCs contribute largely to providing clinical education and mentorship, developing training materials and protocols, and coaching around quality improvement. Notably, as part of the Nepal PPP, GHCs do not participate in direct service delivery, but rather serve in an advisory role, as their primary areas of responsibility involve building the clinical and technical expertise of Nepali providers. As signatories to the Non-Governmental Organization Code of Conduct for Health Systems Strengthening [61,62], *Possible* seeks to adhere to hiring practices that strengthen national healthcare systems, including ‘employing available national expertise, particularly where unemployment of highly qualified nationals abounds’. A critical strategy of the PPP is that non-Nepali providers add value by building national capacity, and not by providing care themselves. Indeed, by leveraging the MoH and *Possible*’s existing healthcare delivery PPP, we have avoided the challenges in coordinating multiple, disjointed expatriate clinicians that so often happen in the healthcare sector [37,42].

GHCs additionally support implementation research efforts through *Possible*’s research arm, the Healthcare Systems Design Group [63], training around digital systems, and co-authorship on academic publications. Here we view the contribution of co-writing manuscripts not solely from the lens of GHC promotion. Rather, we consider disseminating findings central to the principle of beneficence in implementation research, as well as important for furthering equitable opportunities for LMIC authorship [64].

### Reciprocity in professional development opportunities

A central consideration – and perhaps the biggest challenge – for GHAPs is seeking equity and reciprocity. While one might expect a ‘get one, take one’ approach [27], this is typically not the case. Indeed, there are great asymmetries in the distribution of opportunity and benefit involved in GHAPs. The flow of opportunity, mobility, as well as social and professional capital, trends largely to participants and institutions from wealthier countries. As part of the partnership, *Possible* nominates qualified Nepali staff to receive intensive global health training in the US, structured mentorship through HEAL, and the opportunity for fully sponsored enrollment into master’s degree programs in the US and UK as site fellows. HEAL also facilitates site exchanges at other international partner sites by including both site and rotating fellows of the Nepal partnership to exchange knowledge with their counterparts around the world. Fellowships are not solely available to doctors, and HEAL has provided fellowships to pharmacists, social workers, administrators, and government officials. The current ratio of US rotating fellows to Nepali site fellows is 3:2. The ratio of rotating to site fellows across all HEAL partner sites, which includes domestic US sites serving marginalized communities, is 1:1.

Reciprocity also comes in the form of financing, acknowledging the extreme disparities involved in HIC academic medical centers and LMIC organizations. To ensure sustainability, most funding comes from the US academic institutions affiliated with HEAL. Some organizational costs for onboarding, travel, lodging, and administrative support are born by *Possible*, which budgets US$8,000 per Fellow per year.

## Framework and measures for impact

The effectiveness of GHAPs is notoriously difficult to quantify [2], as are the potential unintended consequences and costs of getting these partnerships wrong. In Table 1, we organize the key components of this partnership model within the THET Principles of Partnership framework [39]. THET describes eight principles for partnerships to be effective: they should be (1) strategic; (2) harmonized and aligned; (3) effective and sustainable; (4) respectful and reciprocal; (5) organized and accountable; (6) responsible; (7) flexible, resourceful, and innovative; and (8) committed to joint learning. We find this framework useful for assessing the quality of engagements at several levels, including personal, organizational, healthcare systems, and population.

We also qualitatively measure partnership success by asking the Nepali clinical leadership team at *Possible* the following question: Am I (and/or my team) being supported in my role by GHC partners? As the quality improvement mantra states, ‘you cannot improve what you do not measure’, and this maxim applies here. Yet, thus far, we have struggled to measure and track this precisely as it applies to our Nepal partnership. We have developed an instrument to evaluate the success and satisfaction of GHCs with the Nepal partnership, as shown in Supplemental File 3. We will continue efforts to quantitatively and qualitatively measure the success of partnerships and search for novel metrics to reflect their value, in order to facilitate ongoing iterations and improvements. Ideally, we would identify a way to institutionalize an iteration on this THET Principles of Partnership framework for internal improvement and partnership-building; outside of this paper, however, we have yet to do so.

## Limitations of the partnership model

There are several limitations to the model. The investment of fully integrating academic partners into the organization is time- and labor-intensive. The cost is lowered in *Possible*’s organizational structure by strong existing and templated workflow practices, as well as a clear delineation of roles. Thus, other organizations may find this process more burdensome.

While we aim to build equity into our partnership, the inequities that exist between team members from HICs and LMICs are largely indelible. We would like to better bring in other voices throughout the organization, particularly among those who do not speak English and are not among the leadership team. This will be an important next step in refining our partnership. One strategy would be to conduct focus group discussions and key informant interviews with Nepali providers to better understand strengths and challenges of the partnership.

While the partnership works to address career development needs of GHCs, the barriers are not mitigated entirely, in large part because GHC career paths are often difficult to define. HEAL is considering a larger study of the long-term career outcomes of GHCs within both this partnership and other sites. That longitudinal analysis will bring some evidence to bear on the questions of the value proposition to the GHCs.

Most importantly, global healthcare delivery demands tools and perspectives from multiple disciplines. As one example, the emergent field of implementation research, also called implementation science – broadly defined as the application of rigorous mixed methods to the adaptation, testing, and scaling of innovative, effective interventions with the goal of closing the ‘know-do gap’ and shaping broader policy environments – brings together the gamut of disciplines concerned with improving health and healthcare systems: from medicine and the allied health sciences, to demography, operations research, political science, and medical anthropology [65–70]. While we focus here on clinician partnerships, we have not yet figured out how to deliver responsibly on other forms of engagement, and this remains a weakness because of the immense importance of other disciplinary approaches towards understanding and addressing plural approaches to health and healing.

Our Nepal partnership experiences also may have limited generalizability, as this is one partnership in a specific context with a unique set of partners. Challenges inherent to measuring the success of academic partnerships remain significant here. We apply the THET Principles of Partnership framework as a heuristic to partially mitigate this concern, though it does not address efforts to compare the effectiveness of different partnership models. We focus on several aspects of the partnership model we feel important, although it is likely there are numerous intangible aspects which remain unmentioned in this paper.

## Conclusions

It is challenging for host institutions to leverage GHCs in effective and equitable ways, and similarly challenging to identify viable pathways for clinicians to do this work responsibly. Here we provide a descriptive account of one approach, highlighting key structural and relational components that have helped to mitigate the challenges of GHAPs. We acknowledge that the concept of true partnership is elusive and is more accurately realized as part of a continuous process rather than an achieved state.

Insights from our approach include: the importance of prioritizing targeted engagements with academic medical centers; the need for efficient organizational work-flow practices to minimize integration cost; and the ideal of prioritizing national and host institution goals, while simultaneously addressing the career development needs of GHCs. The complementary roles of managers and faculty coaches deserves repeating as a key aspect of this model. The direct manager internal to the organization addresses GHC accountability to and alignment with organizational priorities, aimed at supporting rather than undermining local providers and leadership capacity. GHC relationships with faculty coaches address networking, research, manuscripts, funding sources, and career development. Managers and coaches are, thus, able to leverage each other’s skills and perspectives to bring value to the work of *Possible* and the GHC. Building an equitable partnership will take time, flexibility, and iteration. If we are to disrupt traditional partnership models where the locus of power, resources, and opportunities trends asymmetrically towards HICs and GHCs, we must pay special attention to deep and persistent inequities.

While the approach we take here is unique to our organizations and setting, this descriptive account will ideally help other organizations create new partnerships and refine existing ones that promote strengthening national capacity and public sector healthcare systems.

## Supplementary Material

Supplemental Table 2

Supplemental Table 1 1

Supplemental Data 3

Supplemental Data 2

Supplemental Data 1

## Paper context

The benefits of global health academic partnerships include promotion of research, advocacy, and medical education. However, such partnerships may undermine local priorities and perpetuate exploitative relationships. Guidelines have been developed to facilitate the creation of effective, equitable partnerships. This paper explores the practicalities of a concrete partnership to demonstrate how guiding principles can be translated into tangible models. Moving forward, the inclusion of additional perspectives, and development of robust partnership measurement tools would add value.

## References

[1] Crane J. Scrambling for Africa? Universities and global health. Lancet. 2011;377:1388–9. 21074254 10.1016/S0140-6736(10)61920-4

[2] Kelly E, Doyle V, Weakliam D, et al A rapid evidence review on the effectiveness of institutional health partnerships. Global Health. 2015;11:48. 26666356 10.1186/s12992-015-0133-9 PMC4678480

[3] Elmusharaf K, Tahir H, O’Donovan D, et al From local to global: a qualitative review of the multi-leveled impact of a multi-country health research capacity development partnership on maternal health in Sudan. Global Health. 2016;12:20. 27184907 10.1186/s12992-016-0153-0 PMC4869333

[4] Networking and coalition building: lessons from a Latin American Network. COHRED learning brief. Geneva: Council on Health Research for Development (COHRED); 2000.

[5] McCoy D, Mwansambo C, Costello A, et al Academic partnerships between rich and poor countries. Lancet. 2008;371:1055–1057. 18374827 10.1016/S0140-6736(08)60466-3

[6] Costello A, Zumla A Moving to research partnerships in developing countries. BMJ. 2000;321:827. 10.1136/bmj.321.7264.827 PMC1118627 11009530

[7] Lansang MA, Dennis R Building capacity in health research in the developing world. Bull World Health Org. 2004;82:764–770. PMC2623028 15643798

[8] Kim JYMDP, Evans TGD Redefining the measure of medical education: harnessing the transformative potential of MEPI. Acad Med. 2014;89:S29–S31. 25072572 10.1097/ACM.0000000000000344

[9] Farmer P, Rhatigan J Embracing medical education’s global mission. Acad Med. 2016;91:1592–1594. 10.1097/ACM.0000000000001433 27749305

[10] Busse H, Aboneh EA, Tefera G Learning from developing countries in strengthening health systems: an evaluation of personal and professional impact among global health volunteers at Addis Ababa University’s Tikur Anbessa Specialized Hospital (Ethiopia). Global Health. 2014;10:64. 25190076 10.1186/s12992-014-0064-x PMC4172777

[11] Syed SB, Dadwal V, Rutter P, et al Developed-developing country partnerships: benefits to developed countries? Global Health. 2012;8:17. 10.1186/1744-8603-8-17 PMC3459713 22709651

[12] Bhattacharyya O, Khor S, McGahan A, et al Innovative health service delivery models in low and middle income countries – what can we learn from the private sector? Health Res Policy Syst. 2010;8:24. 20630108 10.1186/1478-4505-8-24 PMC3236300

[13] Kulasabanathan K, Harris M, Bhatti Y, et al Do international health partnerships contribute to reverse innovation? A mixed-methods study of THET-supported partnerships in the UK. Global Health. 2017;13:25. 10.1186/s12992-017-0248-2 PMC5395771 28420405

[14] Gupta AR, Wells CK, Horwitz RI, et al The international health program: the fifteen-year experience with Yale University’s internal medicine residency program. Am J Trop Med Hyg. 1999;61:1019–1023. 10674689 10.4269/ajtmh.1999.61.1019

[15] Bazemore AW, Henein M, Goldenhar LM, et al The effect of offering international health training opportunities on family medicine residency recruiting. Fam Med. 2007;39:255–260. 17401769

[16] Migone C, Weakliam D The benefits and costs to the Irish health service of health partnerships with low- and middle-income countries. In: Evidence, effectiveness & impact “THET conference”. London; 2016.

[17] Corbin JH, Mittelmark MB, Lie GT Mapping synergy and antagony in north–south partnerships for health: a case study of the Tanzanian women’s NGO KIWAKKUKI. Health Promot Int. 2013;28:51–60. 22180447 10.1093/heapro/dar092 PMC3566659

[18] Johanna TC Unequal ‘partners’. AIDS, academia, and the rise of global health. Behemoth J Civil. 2010;03:78–97.

[19] Wildridge V, Childs S, Cawthra L, et al How to create successful partnerships—a review of the literature. Health Inf Libraries J. 2004;21:3–19. 10.1111/j.1740-3324.2004.00497.x 15186286

[20] Kraeker CMDFMDTM, Chandler CMP “We learn from them, they learn from us”: global health experiences and host perceptions of visiting health care professionals. Acad Med. 2013;88:483–487. 23425985 10.1097/ACM.0b013e3182857b8a

[21] Green T, Green H, Scandlyn J, et al Perceptions of short-term medical volunteer work: a qualitative study in Guatemala. Global Health. 2009;5:4. 19245698 10.1186/1744-8603-5-4 PMC2662818

[22] Geissler PW, Okwaro F Developing world: discuss inequality. Nature. 2014;513:303. 25230632 10.1038/513303a

[23] Ong A, Collier SJ Global assemblages: technology, politics, and ethics as anthropological problems. Malden (MA): Blackwell Pub; 2005.

[24] Berry NS Did we do good? NGOs, conflicts of interest and the evaluation of short-term medical missions in Sololá, Guatemala. Soc Sci Med. 2014;120:344–351. 24834868 10.1016/j.socscimed.2014.05.006

[25] Roche S, Ketheeswaran P, Wirtz V Medicine without borders: a literature review of short-term international medical missions. Lancet Glob Health. 2015;3:S12.

[26] Banatvala N, Doyal L Knowing when to say “no” on the student elective. BMJ. 1998;316:1404. 9572746 10.1136/bmj.316.7142.1404 PMC1113113

[27] Erikson SL, Wendland C Exclusionary practice: medical schools and global health clinical electives. Student BMJ. 2014;22.

[28] Grennan T A wolf in sheep’s clothing? A closer look at medical tourism. McMaster Univ Med J. 2003;1:50–54.

[29] McLennan S Medical voluntourism in Honduras: ‘helping’ the poor? Prog Dev Stud. 2014;14:163–179.

[30] Morgan M Another view of “humanitarian ventures” and “fistula tourism”. Int Urogynecol J. 2007;18:705–707. 10.1007/s00192-007-0309-9 17252312

[31] Scarisbrick G Medical tourism should be banned. BMJ Career Focus. 2002;324:S7.

[32] Smith K The problematization of medical tourism: a critique of neoliberalism. Dev World Bioeth. 2012;12:1–8. 22420447 10.1111/j.1471-8847.2012.00318.x

[33] Federico SG, Zachar PA, Oravec CM, et al A successful international child health elective: the university of colorado department of pediatrics’ experience. Arch Pediatr Adolesc Med. 2006;160:191–196. 16461877 10.1001/archpedi.160.2.191

[34] Crump JA, Sugarman J Ethics and best practice guidelines for training experiences in global health. Am J Trop Med Hyg. 2010;83:1178–1182. 21118918 10.4269/ajtmh.2010.10-0527 PMC2990028

[35] Larkan F, Uduma O, Lawal SA, et al Developing a framework for successful research partnerships in global health. Global Health. 2016;12:17. 27154550 10.1186/s12992-016-0152-1 PMC4859962

[36] Loh LC, Cherniak W, Dreifuss BA, et al Short term global health experiences and local partnership models: a framework. Global Health. 2015;11:50. 26684302 10.1186/s12992-015-0135-7 PMC4683927

[37] Provenzano AM, Graber LK, Elansary M, et al Short-term global health research projects by US medical students: ethical challenges for partnerships. Am J Trop Med Hyg. 2010;83:211–214. 20682858 10.4269/ajtmh.2010.09-0692 PMC2911161

[38] Melby MKPMMA, Loh LCMDMPH, Evert JMD, et al Beyond medical “missions” to impact-driven short-term experiences in global health (STEGHs): ethical principles to optimize community benefit and learner experience. Acad Med. 2016;91:633–638. 26630608 10.1097/ACM.0000000000001009

[39] THET principles of partnership: Tropical Health and Education Trust. [cited 2017 3 1]. Available from: http://www.thet.org/pops/principles-of-partnership

[40] Acharya B, Maru D, Schwarz R, et al Partnerships in mental healthcare service delivery in low-resource settings: developing an innovative network in rural Nepal. Global Health. 2017;13:2. 28086925 10.1186/s12992-016-0226-0 PMC5237195

[41] Swarr AL, Nagar R Critical transnational feminist praxis. Albany (NY): State University of New York Press; 2010.

[42] Citrin D The anatomy of ephemeral health care: “health camps” and short-term medical voluntourism in remote nepal. Stud Nepali Hist Soc. 2010;15:27–72.

[43] Koplan J, Bond TC, Merson M, et al Towards a common definition of global health. Lancet. 2009;373:1993–1995. 19493564 10.1016/S0140-6736(09)60332-9 PMC9905260

[44] Maselli D, Lys J-A, Schmid J Improving impacts of research partnerships. Berne: Swiss Commission for Research Partnerships with Developing Countries, KFPE; 2004.

[45] Kasper J, Bajunirwe F Brain drain in sub-Saharan Africa: contributing factors, potential remedies and the role of academic medical centres. Arch Dis Child. 2012;97:973. 22962319 10.1136/archdischild-2012-301900

[46] Binagwaho A, Kyamanywa P, Farmer PE, et al The human resources for health program in Rwanda—a new partnership. N Engl J Med. 2013;369:2054–2059. 24256385 10.1056/NEJMsr1302176

[47] Panosian C, Coates TJ The new medical “missionaries”—grooming the next generation of global health workers. N Engl J Med. 2006;354:1771–1773. 16641393 10.1056/NEJMp068035

[48] Greysen SR, Richards AK, Coupet S, et al Global health experiences of U.S. physicians: a mixed methods survey of clinician-researchers and health policy leaders. Global Health. 2013;9:19. 23663501 10.1186/1744-8603-9-19 PMC3655883

[49] Kolars JCMD, Cahill KMPH, Donkor PM, et al Perspective: partnering for medical education in sub-Saharan Africa: seeking the evidence for effective collaborations. Acad Med. 2012;87:216–220. 22189887 10.1097/ACM.0b013e31823ede39

[50] McCarthy AE, Petrosoniak A, Varpio L The complex relationships involved in global health: a qualitative description. BMC Med Educ. 2013;13:136. 24090069 10.1186/1472-6920-13-136 PMC3819699

[51] Umoren RA, Einterz RM, Litzelman DK, et al Fostering reciprocity in global health partnerships through a structured, hands-on experience for visiting postgraduate medical trainees. J Grad Med Educ. 2014;6:320–325. 10.4300/JGME-D-13-00247.1 PMC4054735 24949140

[52] Kumwenda B, Dowell J, Daniels K, et al Medical electives in sub-Saharan Africa: a host perspective. Med Educ. 2015;49:623–633. 25989410 10.1111/medu.12727

[53] Citrin DM “Paul Farmer made me do it”: a qualitative study of short-term medical volunteer work in remote Nepal [thesis (M.P.H.)]. University of Washington; 2011.

[54] Palazuelos DMDMPH, Dhillon RMD Addressing the “global health tax” and “wild cards”: practical challenges to building academic careers in global health. Acad Med. 2016;91:30–35. 26244256 10.1097/ACM.0000000000000845 PMC4885528

[55] Shoeb M, Le P, Greysen SR Global health hospitalists: the fastest growing specialty’s newest niche. J Hosp Med. 2013;8:162–163. 23418158 10.1002/jhm.2017

[56] Wendland CL Moral maps and medical imaginaries: clinical tourism at Malawi’s College of Medicine. Am Anthropol. 2012;114:108–122. 22662357 10.1111/j.1548-1433.2011.01400.x

[57] Maru D, Upreti SR Health affairs. 2015 [ cited 2017]. Available from: http://healthaffairs.org/blog/2015/07/20/the-high-costs-of-nepals-fee-for-service-approach-to-health-care/

[58] Biehl J, Petryna A When people come first: critical studies in global health. Princeton (NJ): Princeton University Press; 2013.

[59] Pfeiffer J, Chapman R Anthropological perspectives on structural adjustment and public health. Annu Rev Anthropol. 2010;39:149–165.

[60] Zimmerman M, Shakya R, Pokhrel BM, et al Medical students’ characteristics as predictors of career practice location: retrospective cohort study tracking graduates of Nepal’s first medical college. BMJ. 2012;345:e4826. 10.1136/bmj.e4826 PMC3419272 22893566

[61] The NGO code of conduct for health systems strengthening: health alliance international. [cited 2017 3 1]. Available from: http://ngocodeofconduct.org/

[62] Pfeiffer J, Johnson W, Fort M, et al Strengthening health systems in poor countries: a code of conduct for nongovernmental organizations. Am J Public Health. 2008;98:2134–2140. 18923125 10.2105/AJPH.2007.125989 PMC2636539

[63] Healthcare systems design group: implementation research for patient-centered design: possible. 2015 [cited 2017 3 1]. Available from: www.hsdg.partners.org

[64] Kohrt BA, Upadhaya N, Luitel NP, et al Authorship in global mental health research: recommendations for collaborative approaches to writing and publishing. Ann Glob Health. 2014;80:134–142. 24976552 10.1016/j.aogh.2014.04.007 PMC4155487

[65] Remme JH, Adam T, Becerra-Posada F, et al Defining research to improve health systems. PLoS Med. 2010;7:e1001000. 21124816 10.1371/journal.pmed.1001000 PMC2993153

[66] Schackman BRP Implementation science for the prevention and treatment of HIV/AIDS. JAIDS. 2010;55:S27–S31. Prevention and Treatment of HIV/AIDS Among Drug-Using: AGlobal Perspective. 21045596 10.1097/QAI.0b013e3181f9c1da PMC3058234

[67] World Health Organization. Implementation research toolkit: facilitators’ guide Geneva: World Health Organization on behalf of the Special Programme for Research and Training in Tropical Diseases; 2014.

[68] World Health Organization Report from the ministerial summit on health research: identify challenges, inform actions, correct inequities. Geneva: World Health Organization and Government of Mexico; 2005.

[69] World Health Organization Bridging the “know–do” gap: meeting on knowledge translation in global health Geneva: World Health Organization; 2006.

[70] Pakenham-Walsh N Learning from one another to bridge the “know-do gap”. BMJ. 2004;329:1189.

