# Supplementary material for: Power, potential, and pitfalls in global health academic partnerships: review and reflections on an approach in Nepal
Source: Glob Health Action. 2017 Sep 15;10(1):1367161. doi: 10.1080/16549716.2017.1367161 (PMC5645653; doi:10.1080/16549716.2017.1367161)
Supplement: Supplemental Table 2 [file ZGHA_A_1367161_SM5385.docx]

Supplemental Table 2. Division of labor plan between manager and faculty coach

| Domain | Manager Role | Faculty Coach Role |
| --- | --- | --- |
| *Identification of Expertise/*  *Resource Needs* | Communicates gaps in resources, particularly during annual strategy sessions. | Assigned based on expertise and resource needs for the clinician. |
| *Recruitment & Hiring Process* | Given the unique aspects of recruiting and partnering with academic centers, the Chief of Staff is the hiring lead for academic faculty, fellows, and trainees. The manager, if identified prior to the engagement process, has a veto say in hiring. But much of the vetting/management is delegated to the Chief of Staff. | Called upon during the interview process, though not necessarily. |
| *Organizational Onboarding* | Similar to other full-time employees, onboarding is conducted by the People Operations team. The manager conducts the management-specific parts. | Identifies career goals/needs and explains the nuances of interfacing with academic medical centers. |
| *Organizational Culture & Management Tools* | Drives and reinforces organizational culture including lexicon, readings, task/inbox management, and partnership management. The manager leverages the People Operations team for support—that is, the manager should not feel pressure to coaching/policing the academic clinician in work-flow issues (there will be substantial needs), but, when issues are identified, to notify People Operations team to assist. | Assists in this role and reinforces organizational culture at all opportunities. Compassionately encourages use of the People Operations team as a resource to adjust ingrained habits created by workflow practices of academic and healthcare organizations that do not align with organizational culture. |
| *Weekly Learning & Performance Reviews* | Holds weekly 1:1 and monthly reflection sessions, develops Areas of Responsibilities and Objectives & Key Results, and conducts performance reviews. | Schedules monthly coaching meetings, which are public in project management system. Assists with drafting Areas of Responsibilities and Objectives & Key Results as needed. |
| *Project Planning* | Drives this process through close alignment with programmatic goals and organizational strategy. | Assists in this role in consultation with and deference to the manager. |
| *Academic Manuscript Writing* | Provides feedback, edits drafts, and identifies mode/style of write-up relevant to programs and program teams. The manager will typically be a co-author on manuscripts due to their participation in the program being written about. | Guides team member by identifying appropriate journals, reviewing and editing manuscript, and identifying co- authors. Supports and assists with input from manager when appropriate. |
| *Time Management* | Identifies priorities and assists with triaging work. Works closely to identify key times for team member to spend in Nepal. | Assists with negotiating clinical time at home institution and aims to overlap with team member during Nepal trips. |
| *Research Support* | Identifies strategic areas for research based on programmatic priorities as well as the relevance, acceptability, and feasibility of research in the local communities in Nepal. | Ensures close collaboration with research team members, including driving research protocol development and IRB submission processes. |
| *Academic Promotion* | Assists as needed. | Coaches team member in preparing and updating CV and promotion materials as needed. |
| *Grant Writing & Revenue* | Assists as needed. | Coaches team member in identifying funding and revenue sources, and with grantsmanship. |
