# Supplementary material for: Power, potential, and pitfalls in global health academic partnerships: review and reflections on an approach in Nepal
Source: Glob Health Action. 2017 Sep 15;10(1):1367161. doi: 10.1080/16549716.2017.1367161 (PMC5645653; doi:10.1080/16549716.2017.1367161)
Supplement: Supplemental Table 1 1 [file ZGHA_A_1367161_SM5351.docx]

Supplemental Table 1a. HEAL’s fellow selection rubric

| Name | Social Justice/ Vulnerable Populations^1^ | Compassion/  Cultural Competence^2^ | Advocacy^3^ | Career Trajectory^4^ | Teamwork^5^ | Academic/ Clinical Ability^6^ | Flexibility^7^ | Total^a^ |
| --- | --- | --- | --- | --- | --- | --- | --- | --- |
|  | | | | | | | |  |

*Questions:*

1. Do they have a genuine commitment to social justice and serving vulnerable populations?
2. Do they demonstrate genuine compassion and the cultural competence to serve vulnerable populations, both domestically and abroad?
3. What is their potential to be a strong advocate?
4. What is their likelihood of a career trajectory in global health?
5. What is their approach to teamwork? What are their inter-professional skills?
6. How strong is their clinical aptitude and clinical ability?
7. How flexible are they?

*Scoring:*

1. Each question receives a score from 1-6.

Supplemental Table 1b. Possible’s global health clinician selection rubric

| First Round, Application Review: Qualifications/Preliminary Fit | | | | | | | | | | | | | | | |
| --- | --- | --- | --- | --- | --- | --- | --- | --- | --- | --- | --- | --- | --- | --- | --- |
| Name | **South Asia**^1^ | **Career Trajectory**^2^ | | **Quality Improvement**^3^ | | **Teaching Experience**^4^ | | **Health Systems**^5^ | | **Rural Experience**^6^ | | **Fit**^7^ | | **Reflective**^8^ | **Total**^a^ |
|  |  |  | |  | |  | |  | |  | |  | |  |  |
| Second Round, Interview: Organization Culture Fit | | | | | | | | | | | | | | | |
| Name | **Nepal**^9^ | | **Career Trajectory**^10^ | | **Areas of Responsibility**^11^ | | **Teamwork**^12^ | | **Work Tools**^13^ | | **Culture**^14^ | | **Articulative**^15^ | | **Total**^a^ |
|  |  | |  | |  | |  | |  | |  | |  | |  |

*Questions:*

1. Do they have experience or interest in South Asia?
2. Do they articulate their role in global health? Is it in alignment with our organization’s mission/values/goals/identity?
3. Do they have experience with quality improvement projects?
4. Do they have experience teaching clinicians and other practitioners/implementers abroad?
5. Do they mention interest/experience with government, health systems strengthening, and standard operating protocols?
6. Have they lived or worked (or are interested) in working in a rural area?
7. Do they have any interests or experiences that would be a unique fit for our programs? E.g. electronic medical record system development, community health, or implementation research?
8. Do they demonstrate a sense of critical self-awareness in their personal statement?
9. Why are they interested in Nepal? Is this motivation in alignment with our organization’s goals/mission?
10. Do they see themselves involved in South Asia long-term? What is their interest in global health long term? Is this aligned with our organization’s goals/mission?
11. Do our site-specific areas of responsibilities resonate with them? What are their strengths/weaknesses in terms of these responsibilities?
12. How do they work with others? Do they seem like a good fit for working with our team?
13. Do they have a strategy for organizing their work? Is it specific? Do they seem like a good fit for our project management style and system?
14. How does our for-impact culture code resonate with them? Did they take the time to review and critique it?
15. Are they articulate, critical, passionate, and interested about our work and about our organization?

*Scoring:*

1. Each question receives a score from 1-3. Totals from first and second round are then summed.
