# Supplementary material for: Power, potential, and pitfalls in global health academic partnerships: review and reflections on an approach in Nepal
Source: Glob Health Action. 2017 Sep 15;10(1):1367161. doi: 10.1080/16549716.2017.1367161 (PMC5645653; doi:10.1080/16549716.2017.1367161)
Supplement: Supplemental Data 3 [file ZGHA_A_1367161_SM5127.pdf]

Supplemental File 3. Global Health Clinician site evaluation

Overview
We aim to provide a meaningful, structured placement for residents and other academic clinician implementers. We also believe that you cannot improve what you don't measure. This exit survey is comprised of open-ended questions intended to elicit a deeper understanding of trainee and academic faculty members' experience with Possible in order to improve these engagements: for future fellows, for the organization, and for the people we directly serve through our work. These questions together provide an evaluation framework intended to help assess the impact of the engagement at several levels: personal, organizational, healthcare system, and population level.

Instructions
This evaluation should be conducted by the fellow's manager or coach in person, or through a video conference call if an in-person option is not possible.
Approach this evaluation like a semi-structured interview; that is, (i) let these questions guide an open-ended conversation, allowing for small detours into other stories and areas of reflection that may be useful, but at the same time staying focused on the emergent insights we're after; (ii) in addition, feel free to treat this instrument as a question "bank," from which you can choose the most appropriate questions per individual cases. Some of the topics will likely overlap, and therefore you will likely not ask all of these questions. Take short notes as needed throughout the interview, but do not let it detract from the active listening needed to help the fellow reflect on the bring back insight to our program directors/organization.

When finished, write a brief summary, providing a few bullet points for each section as key take-aways for the organization and complete other post-survey tasks.

Preparation:

- ☐ Were you able to obtain all the information you needed about Possible as a placement site to help you make an informed decision? How might we improve this process?

- ☐ Were you contacted by someone from Possible in a timely manner shortly after the engagement was finalized? What other forms of communication or background information would be useful to other incoming residents/fellows?

- ☐ Did you feel Possible's on-boarding process left you with a thorough and clear understanding of how you would interface with various team (members) during your placement? If yes, which parts specifically where of high value? How might we improve upon this process?

- ☐ Did you feel Possible's on-boarding process left you with a thorough and clear understanding of organizational work culture? Please explain

- ☐ Was there clarity in expectations of your primary work responsibilities and tasks before arriving In Nepal? Please comment.

- ☐ Did you have a clear picture of what your day-to-day work and non-work life would look like

before arriving in Nepal?

- ☐ How else can we improve our preparation and orientation for future fellows? Please be as specific as possible.

Competencies

- ☐ Did the placement advance your understanding of the structural determinants of health and health disparities? Please explain.

- ☐ Did the placement provide you with exposure to (clinical) work and training that was culturally humble? Were you exposed to conversations/experiences that prompted you to reflect inward and promote cultural humility? Did the training you receive here seek to further these reflections?

Cultural humility implies a willingness to engage in constant reflection, and to examine situations from as many perspectives as possible. In distinction to the concept of 'cultural competency' - the flawed notion that one can acquire mastery or expertise in "knowing" cultures - cultural humility involves acknowledging the limits of one's knowledge and capacities in complex settings. This is particularly important for clinicians, were the concept of "achieving competency" is so engrained and sought after.

This question really gets at whether, as an organization, we have built a team culture that perpetuates humility in the face of complexity: an awareness of multiple geo-social positions within our own team, and how these asymmetrical power relationships and dynamics inevitably shape the work that we do, and the (expectations of) care we provide.

Conversations/Experiences:

- ☐ Did your placement shape your thinking on population health and equity? Please explain.

- ☐ To what extent do you feel you were able to effectively integrate into the local healthcare delivery system?

- ☐ At Possible, our foremost maxim is to "solve for the patient." We might say, we aim to solve "with the patient." Describe your ability to actualize on being an advocate for health and wellbeing for patients/the people of Nepal during your placement. What was the impact on you (on these communities)?

- ☐ Describe your primary contributions to the organization's strategic mission in Nepal?

- ☐ Do you feel that you provided or contributed to something enduring within the public sector healthcare system during your time with Possible? Please explain.

This might be e.g. through helping develop or scale Possible's electronic medical record system; working with government partners to bring a pilot health insurance scheme to the districts where Possible works; or training mid-level government providers in advanced respiratory therapy techniques.

- ☐ Did the organization hold you and all its team members to the highest level of ethical conduct in healthcare delivery and implementation science research? Please explain.

- ☐ Where you registered with appropriate council(s)? Did you observe informed consent practiced to the best standard possible when appropriate?

- ☐ Were you able to engage in research that both furthered the strategic mission of Possible and furthered your professional and academic objectives?

- ☐ Were you able to engage in work that pushed for equity and quality in public sector healthcare delivery?

Management & Mentorship:

- ☐ Were you given clear tasks by your manager that were consistent with your particular skills and interests? Please explain.

- ☐ Were you engaged in work that both leveraged your areas of expertise and allowed you to pursue professional interests? Please explain.

- ☐ Did you and your mentors arrive at realistic and measurable goals for your time working with Possible?

- ☐ To what extent did your manager listen and respond meaningfully to any ideas, feedback, or concerns as they pertained to programs with which you were directly involved during your placement? This can be in terms of data quality, scientific rigor, or research processes.

- ☐ To what extent was your coach able to help you identify funding and revenue sources and assist with grantsmanship, as needed?

- ☐ Were you supported by your coach to be an independent implementer researcher? If so, in what ways?

- ☐ Overall, was there a clear division of labor between your manager and coach? Please comment on each's ability to successfully support you during your placement.

Communication:

- ☐ Did you leave with a clear understanding of expectations around frequency and means of communication with your manager and coach?

- ☐ How would you rate the level of internal team communication? Please explain.

- ☐ Did you leave with a clear understanding of expectations around work load and continued engagement with Possible?

- ☐ We use Asana as our primary project management tool across our organization. Please

describe your experience using it.

Reflection & Recommendation:

- [ ] Do you feel that Possible lived up to the core principles in its for-impact culture code? If yes, can you provide specific examples? If not, what are the areas in which we as an organization fall short?

- [ ] What was the most rewarding part of your placement with Possible? Please explain.

- [ ] What was the most challenging part of your placement with Possible? Please explain.

- [ ] Are there any resources and/or team members that you wish you had more access to during your time with Possible?

- [ ] How would you structure your role differently for the next person? How would you keep it the same? Please explain.

- [ ] Would you recommend Possible as a placement site to others?

- [ ] Please provide any further candid feedback on ways Possible can improve it's systems to deliver on a well-structured, high value global health training/residency placement site.

Post-Survey:

- [ ] [Manager/Coach] Write a brief summary, providing a few bullet points for each section as key take-aways for the organization.

- [ ] [Manager/Coach] Task all relevant team members to review the notes/main summary.

- [ ] [Manager/Coach] Task all other relevant team members to review the notes/main summary. This will include the fellow's direct manager and coach, care delivery and operations program directors, and the Impact team.
