# Supplementary material for: Power, potential, and pitfalls in global health academic partnerships: review and reflections on an approach in Nepal
Source: Glob Health Action. 2017 Sep 15;10(1):1367161. doi: 10.1080/16549716.2017.1367161 (PMC5645653; doi:10.1080/16549716.2017.1367161)
Supplement: Supplemental Data 2 [file ZGHA_A_1367161_SM5033.pdf]

Supplemental File 2. Global health clinician areas of responsibilities

This document details the responsibilities and expectations for the HEAL Fellows' (Global Health Clinician) work with *Possible* and the Healthcare Systems Design, during their time in Nepal.

Management Structure:
Reports to: Medical Director (per district) and Chief Medical Officer
Direct Reports: N/A
Dotted Line Reports: Staff physicians
Strong collaborative link: Community Health Director & Impact Director

Big 3 Responsibilities:
1. Provide strategic guidance and technical advice to *Possible*'s (Medical, Community Health, and Impact teams) leadership on healthcare systems. This includes protocols development, quality improvement initiatives, and the use of electronic tools for patient care.
2. Improve clinical care at Bayalpata Hospital or Charikot Hospital via direct clinical mentorship of providers and didactic teaching sessions.
3. Provide ideas to, feedback on, any research initiatives pertaining to relevant areas of clinical teaching and quality improvement.

Areas of Responsibility (AoRs):
The HEAL Fellows' primary focus is on improving healthcare systems through strategic guidance and technical assistance to hospital, community health, and impact programs leadership.

1. **Provide strategic guidance and technical advice to *Possible*'s (Medical, Community Health, and Impact teams) leadership on healthcare systems. This includes protocols development, quality improvement initiatives, and the use of electronic tools for patient care.**
    a. Assist in operationalizing protocols with healthcare staff, to encourage simple, feasible protocols that are actually followed and used for patient care;
    b. Lead quality improvement initiatives prioritizing practical change, feasible timelines, and clear outcomes/metrics, including iterative PDSA-cycle workflows and communication of progress and results regularly to team members;
    c. Provide structured guidance to the Impact team on the design of electronic medical records as a tool for improved patient care, provider education, and implementation research;
    d. Champion the use of electronic tools such as digital x-ray, Asana, Up-to-Date, and the organizational electronic medical record;
    e. Provide guidance to Charikot Hospital team on the design of community- and health post-based clinical protocols.

2. **Improve clinical care at Bayalpata Hospital or Charikot Hospital via direct clinical mentorship of providers and didactic teaching sessions.**
    a. Provide direct clinical mentorship to health providers at Bayalpata/Charikot Hospital during daily inpatient rounds, outpatient department clinical encounters, and in the Emergency Department;
    b. Spend at least one day per week in the Outpatient Department with each of TB/HIV, mental health, and any other chronic disease-focused providers;
    c. Work with staff physicians to teach non-physician providers (largely health assistants, but others, including nurses and midwives) in structured continuing medical education lectures

that occur each morning, at least once weekly, with ultimate schedule to be determined on a monthly basis by Medical Director;

d. Give guidance to staff physicians in their own conduct of continuing medical education and morbidity & mortality lectures and their mentorship of non-physician providers;
e. Perform teaching in ultrasound diagnosis and X-Ray interpretation;
f. Provide feedback and assist in action plans to the medical director on the inclusive, structured, efficient, and patient-centered nature of inpatient rounds;
g. Provide feedback to the medical director and assist in action plans to the medical director on outpatient department patient flow, evaluation, and counseling.

3. **Provide ideas to, feedback on, any research initiatives pertaining to relevant areas of clinical teaching and quality improvement.**
    a. Submit concerns about the ethical or efficient conduct of research as they pertain to clinical care and education;
    b. Participate in research initiatives as a clinical and operational consultant;
    c. Identify areas of perspective academic pieces on healthcare systems design
    d. Serve as a co-author on any relevant pieces that merit co-authorship, ie in which the Fellow played a clear vision, design or implementation role.
