# Supplementary material for: Power, potential, and pitfalls in global health academic partnerships: review and reflections on an approach in Nepal
Source: Glob Health Action. 2017 Sep 15;10(1):1367161. doi: 10.1080/16549716.2017.1367161 (PMC5645653; doi:10.1080/16549716.2017.1367161)
Supplement: Supplemental Data 1 [file ZGHA_A_1367161_SM4946.pdf]

Supplemental File 1. Global Health Clinician and faculty coach 1:1 meeting agenda

These are designed to be held once per month for up to 60 minutes. The GHC runs the meeting and is responsible for a professional, efficient meeting with actionable follow-up tasks and clear decisions. The faculty coach is responsible for providing targeted, strategic guidance.

Notes on building an agenda and executing on an excellent 1:1 with your faculty coach:

1) Building an agenda that will lead to an excellent meeting is a task in and of itself. I realistically spend 30-60 minutes building my 1:1 agenda with my manager each week, if not more. This is dedicated time allocated 24-48 hours before the meeting to ensure I'm focusing in on the most priority items. It is arranged according to priority, and I'm getting my manager updates on my Objectives & Key Results (OKRs) and the information she needs to be prepared for our agenda in a concise and organized way.

2) Review and finalize the agenda 24-48 hours ahead of time. This is similar but distinct from #1. Typically I add items to the agenda throughout the week to keep track of things I need to discuss. But I still make sure to then spend a dedicated chunk of time reviewing those items, adding to them, subtracting items that are not relevant any longer, and revising the items I have listed several days before that now have updates given events that have transpired in the interim. This process of organization, re-ordering, revising, and pruning of the agenda is critical to ensuring that I am able to create an agenda that focuses on the priorities and that I have supplied my manager the information she needs to be prepared for each item we need to discuss.

3) Use the OKRs threads to provide updates to ensure the discussion focuses only on the high points. Truthfully I do not need to speak with you about 95% of your OKRs on a weekly basis, though I do need updates, and each of us (as employees) need them to keep us accountable. The goal here is to do so in the most efficient way such that we are not taking up our limited 1:1 time discussing items we are both aligned on, and instead focusing in on the items you need my support on, I have questions about, etc. If you can provide bulleted text updates for each OKR in the 48 hours prior to our 1:1, I will always commit to have read through them, and provided any feedback or questions I have. Then we can skip all of that except the points we each want to discuss. This saves a lot of time while also ensuring you remain accountable to your OKRs, that I am in the loop on the progress you are making, and we are using our 1:1 time together to focus on the tension points.

4) If key updates have a description that I can read ahead of time, we can end up skipping through many of them quickly or not even discussing them. As above, I will commit to having read through everything you provide ahead of time. So in the case of quick key updates that are more of a notification rather than a conversation, please give these to me in text format. Then for many of them we can just check them off or discuss very quickly and then move on. Ultimately this gives more time to the priority items you need feedback on and makes your time much more efficient.

5) Order the agenda to ensure the true priorities are at the front. We will almost always go

over or end up having to review the last few items quickly to squeeze them in. So, plan for that and put the true priority items up front so we can be sure you get the input you need most and we have time to spend on your priorities.

6) Review your agenda before the meeting so it is fresh in your mind and you can quickly and efficiently lead the meeting. This goes for any meetings I run; I review my agendas shortly before (or at most 12-24 hours before) the meeting itself to ensure each agenda item is fresh in my head, I have reviewed the finer details of each agenda item, and I have a clear sense of what my deliverables and goals for the meeting are.

Agenda

- [ ] Read "what it means to act like the CEO of my domain"

This applies to all *Possible* team members: What it means to act like CEO of my domain and act like it. The relevance to you as a faculty member is particularly high. This is especially true because you are part-time in two or three worlds, and need to be highly focused and motivated to navigate your teams at *Possible*, at your academic medical center/university, and their clinical teams if you are on service. You have no one single "boss" and it is up to you to drive value across all the domains of your work, and to integrate them. While your manager and faculty coach at *Possible* will provide support, ultimately you have to be the "CEO of your academic and professional career" for this to work, in addition to be the "CEO of your particular clinical/academic/implementation expertise".

Objectives & Key Results:

- [ ] [Insert your quarterly OKRs here]

Monthly Meeting Prep:

- [ ] Schedule monthly 1:1 meeting with manager/faculty coach while in Nepal and as needed when out of Nepal.

I will depend on you to schedule a 1:1 meeting with me each month. It is best to do this by sending me a Google calendar invite at a time that is free on my Google calendar (if you do not know how to view my organizational Google calendar, just ask me and we can review that). Ideally, meetings should be scheduled at least 1 week in advance so that we both have time to review this meeting space and any preparatory notes that we have put into it. Of course, I am also happy to have "ad hoc" conversations with you at any time, based on your needs.

- [ ] Write agenda items 48 hours in advance of the meeting

The agenda will start with reflections and refactoring. Then we will dive into particular agenda items.

- [ ] Review meeting space prior to the meeting Monthly Agenda Items:

- [ ] [First Meeting] Describe your Areas of Responsibilities (AoRs) and goals (10 min)

- [ ] [First Meeting] Discuss current iteration of OKRs (10 min)

☐ [First Meeting] How you present yourself in Nepal (10 min)

How you present yourself is critical to your being effective as a leader and advocate. Just as there is a role for gravitas in clinical medicine (an air of seriousness, calm, and focus essential to convey to families and other providers) and yet no place for arrogance (which demoralizes families and providers), you need to find a balance of presenting yourself as a very serious and highly accomplished physician-leader yet someone open and humble. Here, we can discuss how you present yourself in Nepal in general, if you have questions.

☐ [First Meeting] Describe your current academic position/role, and your ultimate academic goals, if relevant.

☐ What specific progress have you made against your quarterly OKRs?

Please note in comments where relevant. OKRs should be an active space we are visiting quickly each week. Please make comments where relevant each week. This is set as a recurring task for each week as you prepare for our 1:1s. Be as specific as possible when relevant. For example, if your objective is to submit $1,650,000 worth of institutional funding, each week update with the amount we have fully submitted for and update the total to date for the quarter.

Reflection Questions:
These items below are *suggestion questions* to catalyze thought and discussion, but are not mandatory. Complete them however frequently you would like, or never. Whatever is useful for you.

☐ How I'm bringing value to academic global healthcare.

Pretend I am your chair of medicine and you need to convince me of your value in clinical care, teaching, research, and advocacy. In 3 minutes or less, summarize how you are being a leader in academic global healthcare. As Daniel Pink articulates marvelously in "To Sell is Human", the "pitch" should not be a dirty plague for us to avoid but rather a reality of being human for us to embrace. This is especially true in fields, such as ours, where the path is unclear and revenue sources are extremely limited. It is critical that young faculty members embrace who they are, what they do, and, how they are leaders.

☐ One story that was career affirming last month

It is important to frequently be returning to the "why". Only in understanding our own spiritual motivations can we bring good, serious value to the world, and work with others to join our efforts. This can cover any aspect of your work. It may be "I spent an hour delivering patient-centered care for a homeless patient in Boston" or "I guided an MBBS doc through an morbidity and mortality review" or "I worked with my team to deliver compassionate end of life care" or "I submitted an article to PLOS ONE".

☐ One failure I had in the last month:

- [ ] One 'blind spot' I realized in the last month:

- [ ] One toxic or counter-productive thought I've had in the last month:

- [ ] How am I being or not being the CEO of my domain?

- [ ] Feedback or suggestions to Possible to improve your experience:

- [ ] Reflections on your role and goals, and challenges:

Career Development:
*This space is for you to list career-development opportunities, post-HEAL ideas, etc.

- [ ] [Insert most recent CV here]

- [ ] [Insert most recent HEAL IDP here]

- [ ] Cross-site Visits:

- [ ] Nepali language development

Additional Specific Agenda Items:
These can be personal, professional, interpersonal-- anything that you find of value. Most of the meeting is unstructured so that this can be driven by the faculty member.

- [ ] [Insert agenda items here in order of importance]

Action Items from Meeting:

- [ ] [Insert follow-up tasks and action items here]
